# Effect of Antioxidants in Medicinal Products on Intestinal Drug Transporters

**DOI:** 10.3390/pharmaceutics16050647

**Published:** 2024-05-10

**Authors:** Chetan P. Kulkarni, Jia Yang, Megan L. Koleske, Giovanni Lara, Khondoker Alam, Andre Raw, Bhagwant Rege, Liang Zhao, Dongmei Lu, Lei Zhang, Lawrence X. Yu, Robert A. Lionberger, Kathleen M. Giacomini, Deanna L. Kroetz, Sook Wah Yee

**Affiliations:** 1 Department of Bioengineering and Therapeutic Sciences, University of California, San Francisco, CA 94158, USA; 2 Office of Generic Drugs, Center for Drug Evaluation and Research, FDA, Silver Spring, MD 20993, USA; 3 Office of Pharmaceutical Quality, Center for Drug Evaluation and Research, FDA, Silver Spring, MD 20993, USA

**Keywords:** nitrosamine, antioxidants, intestinal transporters, OATP2B1, BCRP, P-gp

## Abstract

The presence of mutagenic and carcinogenic N-nitrosamine impurities in medicinal products poses a safety risk. While incorporating antioxidants in formulations is a potential mitigation strategy, concerns arise regarding their interference with drug absorption by inhibiting intestinal drug transporters. Our study screened thirty antioxidants for inhibitory effects on key intestinal transporters—OATP2B1, P-gp, and BCRP in HEK-293 cells (OATP2B1) or membrane vesicles (P-gp, BCRP) using ^3^H-estrone sulfate, ^3^H-N-methyl quinidine, and ^3^H-CCK8 as substrates, respectively. The screen identified that butylated hydroxyanisole (BHA) and carnosic acid inhibited all three transporters (OATP2B1, P-gp, and BCRP), while ascorbyl palmitate (AP) inhibited OATP2B1 by more than 50%. BHA had IC_50_ values of 71 ± 20 µM, 206 ± 14 µM, and 182 ± 49 µM for OATP2B1, BCRP, and P-gp, respectively. AP exhibited IC_50_ values of 23 ± 10 µM for OATP2B1. The potency of AP and BHA was tested with valsartan, an OATP2B1 substrate, and revealed IC_50_ values of 26 ± 17 µM and 19 ± 11 µM, respectively, in HEK-293-OATP2B1 cells. Comparing IC_50_ values of AP and BHA with estimated intestinal concentrations suggests an unlikely inhibition of intestinal transporters at clinical concentrations of drugs formulated with antioxidants.

## 1. Introduction

Since the discovery of N-nitrosamine impurities in valsartan in 2018 [1], multiple batches of other drugs such as metformin, ranitidine, and varenicline have been found to contain these impurities. The International Council for Harmonisation of Technical Requirements for Pharmaceuticals for Human Use (ICH) M7 has classified N-nitrosamines, which are formed by secondary or tertiary amines in the presence of nitrosating agents and contain a functional N-nitroso group (>N-N=O), as possible carcinogens [2,3]. Upon exposure, N-nitrosamines can cause genotoxic effects, which can affect important cellular pathways [4,5]. There have been instances where impurities have arisen from oxidation of the active pharmaceutical ingredient itself, for example, nitroso-varenicline and nitroso-propranolol [6]. In general, N-nitrosamine impurities in medications pose a mutagenic and carcinogenic risk, affecting patient safety [7]. The US Food and Drug Administration (FDA), the European Medicines Agency (EMA), and other regulatory agencies have issued guidance for recommending the control of N-nitrosamine impurities in human drugs [8,9].

There are several measures that were put in place to mitigate N-nitrosamine impurities in pharmaceuticals [10]. N-nitrosamine formation can occur due to nitrite in excipients and vulnerable amines associated with the drug manufacturing process or the active pharmaceutical ingredient itself. Recent studies show that antioxidants such as ascorbic acid, sodium ascorbate, α-tocopherol, caffeic acid, and ferulic acid can effectively inhibit N-nitrosamine formation in oral solid dosage forms [11,12,13]. The use of amino acids like glycine, lysine, and histidine as inhibitors in solution has also been suggested [12].

While antioxidants are included in oral medicinal products to enhance drug stability, their potential impact on drug absorption by inhibiting intestinal transporters is unknown [14,15,16]. Intestinal transporters, which are crucial for the absorption of many prescription drugs, include P-glycoprotein (P-gp, MDR1, ABCB1), breast cancer resistance protein (BCRP, ABCG2), and organic anion transporting polypeptide 2B1 (OATP2B1, SLCO2B1) [17,18,19]. Numerous clinical studies have shown that inhibition of these transporters can modulate drug bioavailability or exposure. For example, (i) erythromycin, a P-gp inhibitor, increased the bioavailability of the P-gp substrates digoxin and talinolol [20]; (ii) ML753286, a potent BCRP inhibitor, increased exposure to the BCRP substrates sulfasalazine in monkeys [21]; and (iii) grapefruit, apple, and orange juices were reported to reduce exposure to the OATP2B1 drug substrates fexofenadine and aliskiren [22,23]. Our study aimed to understand the potential liabilities of various antioxidants to these intestinal transporters and their impact on drug absorption. A total of 30 antioxidants were screened for their potential to inhibit these transporters. The findings hold significance for generic and new medicinal products, as they are required to demonstrate bioequivalence or comparative bioavailability of the reformulated medicinal product. Introducing an antioxidant into an existing medicinal product constitutes the creation of a new drug formulation. This change generally necessitates a bioequivalence assessment through an in vivo clinical bioequivalence study. However, for certain drugs that meet specific criteria, an in vitro bridging study may support the reformulation [24]. If sufficient evidence suggests that commonly used antioxidants in oral medicinal products do not significantly affect the activity of the intestinal transporters involved in drug absorption, this could serve as one basis for a waiver of the requirement to provide data from a clinical bioequivalence study for the reformulated products.

## 2. Materials and Methods

### 2.1. Oral Antioxidant Library and Reagents

**Figure 1:**
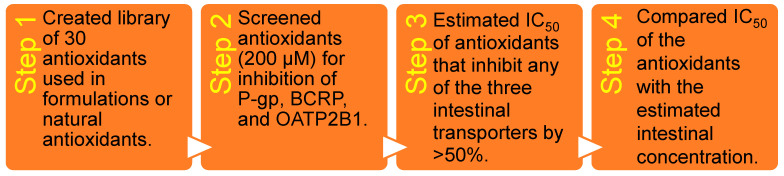
Overview of the screening process.

The University of California San Francisco (UCSF)–Stanford CERSI Excipients Browser (http://excipients.ucsf.bkslab.org/excipients/molecular/?route=oral, access date: 7 May 2022) and the FDA Inactive Ingredient Database (https://www.accessdata.fda.gov/scripts/cder/iig/index.cfm, access date: 7 May 2022) were reviewed to obtain a comprehensive list of 30 antioxidants used in formulations and some known naturally occurring antioxidants. This list was carefully curated by removing duplicates, antioxidants that were no longer in use, those with solubility issues, commercially unavailable ones, and those used in inhalation medicinal products. Additional information on the antioxidants is provided in Table S1. Figure 1 illustrates an overview of the screening process. The initial phase of this study involved identifying antioxidants that demonstrated a significant inhibitory effect (≥50% inhibition) toward the selected transporter function. These antioxidants were then subjected to further analysis through inhibition potency studies (half maximal inhibitory concentration, IC_50_). We chose to screen the antioxidants at high concentrations, specifically 200 µM, to ensure the detection of any putative transport inhibitors. Positive controls cyclosporin A, verapamil, erlotinib, curcumin, elacridar, and Ko-143, and the transporter substrates digoxin and prazosin, were all purchased from Sigma-Aldrich, (St. Louis, MI, USA); the positive control bromosulfophthalein (BSP) was purchased from Pfaltz & Bauer (Waterbury, CT, USA). ^3^H-N-methylquinidine (^3^H-NMQ) and ^3^H-valsartan were purchased from American Radiolabeled Chemicals (St. Louis, MI, USA), and ^3^H-cholecystokinin-8 (^3^H-CCK8), ^3^H-estrone sulfate (^3^H-ES), ^3^H-prazosin, and ^3^H-digoxin were purchased from PerkinElmer (Waltham, MA, USA). P-gp vesicles (ThermoFisher Scientific, #GM0015; Waltham, MA, USA), BCRP vesicles (ThermoFisher Scientific, #GM0008), Isoplate 96-well flat-bottom white plates (PerkinElmer; # 6005070), filtration plates (Millipore, Burlington, MA, USA), Corning BioCoat poly-d-lysine 48-well plate (#356509, Fisher Scientific), Falcon 24 multiwell inserts (#351181, Corning Life Sciences, Corning, NY, USA), 24-well plate (#353047, Corning Life Sciences), Hanks’ balanced salt solution (HBSS; Gibco, Waltham, MA, USA), bovine serum albumin (BSA; Sigma-Aldrich), Dulbecco’s modified Eagle medium (DMEM; Gibco), fetal bovine serum (FBS; Gibco), penicillin and streptomycin (Gibco), glutamine (Gibco), hygromycin B (ThermoFisher Scientific), DMEM with Gluta-Max™ (Gibco), geneticin (Gibco), phosphate-buffered saline (PBS; Gibco), ploy-d-lysine (Gibco), Pierce™ BCA protein assay kit (ThermoFisher Scientific), lucifer yellow (Sigma-Aldrich), EcoLite(+)™ liquid scintillation cocktail (MP biomedical, Burlingame, CA, USA), and scintillation fluid (MicroScint-20 from PerkinElmer) were purchased from the indicated suppliers.

### 2.2. Vesicular Transport Assay

The vesicular transport assay was performed using *Spodoptera frugiperda* 9 (Sf9) membrane vesicles overexpressing human P-gp or BCRP, as reported previously with minor modifications [14,16,25]. In brief, Isoplate 96-well flat-bottom white plates were preincubated with HBSS containing 0.5 mg/mL BSA on an orbital shaker in a 37 °C incubator at 90 rpm for 60 min followed by washing with HBSS. The assay mixture (75 µL/well) contained a probe substrate and either vehicle control, a known inhibitor of P-gp or BCRP, or an antioxidant, in vesicle uptake buffer (50 mM MOPS-Tris, pH 7.0 + 70 mM KCl + 7.5 mM MgCl_2_) prewarmed to 37 °C. After addition of the assay mixture, 5 μL/well (5 μg/μL stock concentration of the vesicles) of vesicles overexpressing human P-gp or BCRP was added. Uptake assays were initiated by adding 20 μL of either 25 mM adenosine triphosphate (ATP) or 25 mM adenosine monophosphate (AMP) to a final concentration of 5 mM. Plates were incubated at 37 °C for 20 min with orbital shaking (90 rpm). After 20 min, the uptake was quenched by adding 150 μL of ice-cold wash buffer (40 mM MOPS-Tris, pH 7.0 + 70 mM KCl) to each well, and the mixture was transferred to a 96-well filtration plate. Vacuum was applied, and the vesicles were washed three times with wash buffer. Vesicles were then lysed with 100 µL of MicroScint-20. The 96-well plates were placed on a shaker at room temperature for 1–2 h prior to measuring the radioactivity by liquid scintillation counting with a MicroBeta^2®^ microplate counter.

### 2.3. Cell Lines and Cell Culture

Human embryonic kidney (HEK) 293 cells overexpressing OATP2B1 (HEK-293-OATP2B1) were cultured and maintained in DMEM supplemented with 10% fetal bovine serum, penicillin (100 U/mL), streptomycin (100 μg/mL), 2 mM glutamine, and 100 µg/mL of hygromycin B at 37 °C in a humidified incubator with 5% CO_2_. MDCK-hMDR1-cMDR1-KO cells, with the endogenous canine MDR1 gene knockout and stable expression of human MDR1, and MDCK-hBCRP-cMDR1-KO cells, with the endogenous canine MDR1 gene knockout and stable expression of human BCRP, were generously provided by Dr. Per Artursson from Uppsala University, along with the corresponding MDCK-cMDR1-KO cells [14,26,27,28]. Cells were cultured in T-25 flasks in DMEM with Gluta-Max™, supplemented with 10% fetal bovine serum, penicillin (100 U/mL), and streptomycin (100 μg/mL). For MDCK cells overexpressing hMDR1, the media were further supplemented with hygromycin B (400 μg/mL), and, for MDCK cells overexpressing hBCRP, the media were supplemented with geneticin (600 μg/mL). The cells were maintained at 37 °C in a humidified incubator with 5% CO_2_. Cells were only utilized through passage 4.

### 2.4. Uptake Transporter Assay

The uptake transporter assay was performed as reported previously with minor modifications [15]. A day before the uptake assay, Isoplate 96-well plates were coated with poly-D-lysine in PBS with calcium and magnesium and washed with PBS. HEK-293-OATP2B1 cells were seeded at a density of 60,000 cells/well. An assay mixture of ^3^H-estrone sulfate, a known inhibitor of OATP2B1, or an antioxidant was prepared and incubated in a 37 °C incubator for 10 min. The cells were washed with ice-cold HBSS three times and incubated with MicroScint fluid on a shaker for 1 h for cell lysis. Radioactivity was measured by liquid scintillation counting with a MicroBeta^2®^ microplate counter. Similar methods were used to assess the inhibition of ^3^H-valsartan-mediated uptake by OATP2B1 using the two antioxidants, AP and BHA. One day before the uptake assay, HEK-293-OATP2B1 cells were seeded in a 48-well poly-d-lysine-coated plate at a density of 120,000 cells/well. On the day of experiment, cells were washed twice with 0.4 mL of HBSS per well and then pre-incubated for 10–20 min in 0.4 mL of HBSS. To assess inhibition, the cells were incubated with uptake buffer (trace amount of ^3^H-valsartan in HBSS) containing either 1% DMSO as negative control, 100 µM erlotinib as positive control, or various concentrations of the antioxidants, AP or BHA. Uptake of ^3^H-valsartan was stopped after 15 min by washing three times with ice-cold HBSS. Cells were lysed in 750 µL of lysis buffer (0.1 N NaOH and 0.1% SDS in double-distilled water) per well while shaking for 60 min. A total of 690 µL of the lysate from each well was then added to 2.5 mL of EcoLite scintillation fluid. The radioactivity was determined on a LS6500 Scintillation Counter (Beckman Coulter). Protein concentration was measured with a Pierce™ BCA protein assay kit. The radioactivity in each well was corrected for protein concentration. All values were determined in triplicate, and the final values are expressed as % uptake relative to negative control (1% DMSO).

### 2.5. Antioxidant Screening and IC_50_ Determination

Antioxidants were screened at a concentration of 200 μM. Screening was carried out in triplicate, and antioxidants that inhibited any of the three transporters by at least 50% were further studied. IC_50_ estimates of the putative inhibitors were obtained by fitting dose–response data (500 µM to 0.03 µM for OATP2B1 and 500 µM to 4 µM for P-gp and BCRP) to the Hill equation by nonlinear regression in GraphPad Prism 9.

### 2.6. Estimation of Intestinal Luminal Concentration (I_gut_) Concentration

*I_gut_* is intestinal luminal concentration estimated as dose/250 mL. The *I_gut_* concentration was estimated using the following equation:

$$I_{gut}=\left[A÷\left(MW\times V\right)\right]\times 10^{6}$$

where *I_gut_* is the estimated intestinal luminal concentration (µM), *A* is the amount of antioxidant in an oral dosage (mg), *MW* is the molecular weight, and *V* is the volume of intestinal fluid, i.e., 250 mL.

### 2.7. Transwell Transport Assay

The transport assay was performed using MDCK-hMDR1-cMDR1-KO, MDCK-cMDR1-KO, and MDCK-hBCRP-cMDR1-KO as reported previously with minor modifications [14,27]. In brief, 133,000 cells were seeded onto a Transwell membrane insert, a semipermeable polyethylene terephthalate membrane. During the differentiation process, the medium volume in the apical chamber was kept at 300 μL, while the basal chamber contained 1000 μL. The cells were allowed to differentiate for 7–9 days at 37 °C in a humidified incubator with 5% CO_2_. Before starting transporter assays, the integrity of the cell monolayer was checked using lucifer yellow permeability assays. Cells were washed twice with HBSS, lucifer yellow (0.1 mg/mL) in HBSS was added to the apical chamber, and the basal chamber was filled with fresh HBSS. The assembled plate was then incubated at 37 °C for 1 h. From the basal chamber, 100 μL samples were collected for fluorescence measurements using GloMax^®^ Explorer with a 475 nm excitation filter and a 500–550 nm emission filter to calculate the percent permeability per well.

To assess the flux of digoxin or prazosin from the basal to apical side, the cells were washed twice with HBSS. In the basal chamber, an HBSS solution containing either digoxin (6.35 nM ^3^H-labeled, final concentration 2.5 μM) or prazosin (43 nM ^3^H-labeled, final concentration 1 μM) was added either with vehicle or a known or putative inhibitor (elacridar at 2 µM for P-gp, Ko143 at 1 µM for BCRP, or BHA at 250, 178, and 62.5 µM). Simultaneously, the same concentration of these inhibitors in HBSS (225 μL) was added to the apical chamber. The plates were incubated at 37 °C with shaking at 200 rpm. Aliquots of 25 μL were collected from the apical compartment at 0, 1, 2, and 4 h, with corresponding replenishment of the apical solution. Samples (25 μL) were also taken from the basal chambers at the start and at the end of each experiment. These samples were used to calculate the disintegrations per minute/picomole ratio. All apical and basal samples were mixed with scintillation fluid (EcoLite(+)™ liquid scintillation cocktail), and their radioactivity was measured using liquid scintillation counting (Beckman LS6500). At the end of the transport assay, a lucifer yellow permeability assay was performed as described earlier. For data analysis, the disintegration per minute counts of digoxin or prazosin from the apical samples were converted to picomoles using radioactivity measured in the substrate solution.

## 3. Results

### 3.1. In Vitro Studies Performed to Assess the Inhibition of 30 Antioxidants on OATP2B1, BCRP, and P-gp

**Figure 2:**
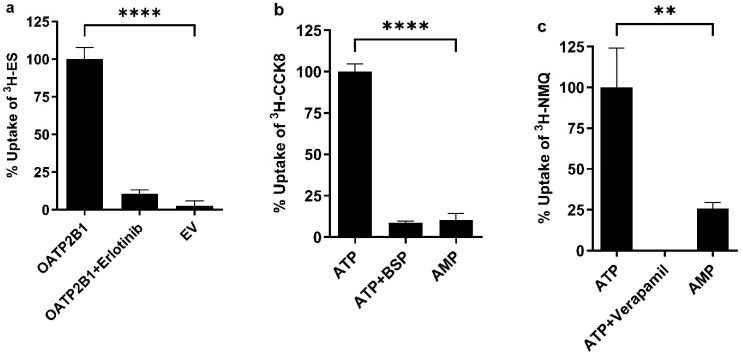
**Validation of OATP2B1, BCRP, and P-gp transporter assays.** (**a**) Uptake of ^3^H-ES by human OATP2B1: HEK-293 cells expressing OATP2B1 (*n* = 6), OATP2B1 in the presence of 20 μM erlotinib (an OATP2B1 inhibitor; *n* = 3), or an empty vector (*n* = 12) were incubated with ^3^H-ES at 37 °C for 10 min. (**b**) Uptake of ^3^H-CCK8 by human BCRP: BCRP membrane vesicles (25 μg/well) were incubated with ^3^H-CCK8 with either 5 mM ATP (*n* = 6), 5 mM ATP with 200 μM BSP (*n* = 3), or 5 mM AMP (*n* = 3) at 37 °C for 20 min. (**c**) Uptake of ^3^H-NMQ by human P-gp: P-gp membrane vesicles (25 μg/well) were incubated with ^3^H-NMQ in the presence of 5 mM ATP (*n* = 6), 5 mM ATP with 200 μM verapamil (*n* = 3), or 5 mM AMP (*n* = 3) at 37 °C for 20 min. Transport in the absence of inhibitors is expressed as 100% and each value represents the mean ± SD; a two-tailed *t*-test was applied *p* < 0.0001: ****; *p* < 0.01: **.

In this study, the probe substrates ^3^H-ES, ^3^H-CCK8, and ^3^H-NMQ were used to measure the activities of OATP2B1, BCRP, and P-gp transporters, respectively [15,16,25,29]. As shown in Figure 2, the uptake of the radioactive probe substrate in human BCRP or P-gp membrane vesicles was significantly higher in the presence of ATP compared to AMP: 9.8 times higher for BCRP and 3.9 times higher for P-gp. In addition, the known inhibitors, BSP (for BCRP), and verapamil (for P-gp) significantly reduced the uptake of these probe substrates, confirming the reliability of our assay in measuring transporter-mediated uptake in membrane vesicles. Notably, ^3^H-NMQ exhibited significant adsorption on the filtration plate (Figure S1), unlike other radioactive probe substrates. In further experiments, as shown in Figure 2, the uptake of ^3^H-ES in HEK-293 cells expressing OATP2B1 was 37.6 times greater than in cells expressing the empty vector (EV). This uptake was significantly inhibited by erlotinib, a known OATP2B1 inhibitor, indicating the suitability of this model substrate for assessing OATP2B1-mediated transport. The scintillation fluid (MicroScint-20) used in this screening assay was not compatible with BCA protein reagents, so we were unable to normalize each well individually by protein concentration. To assess the variance in protein concentration per well, we conducted radioligand uptake using another plate with identical wash steps and found consistent protein concentrations across wells (Figure S2).

**Figure 3:**
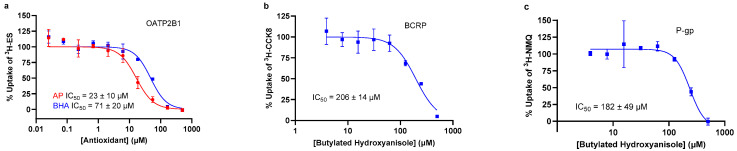
**Concentration–response relationships for inhibition of intestinal transporters by AP and BHA.** (**a**) AP and BHA inhibition of OATP2B1; (**b**) BHA inhibition of BCRP; (**c**) BHA inhibition of P-gp. Reported IC_50_s are the mean ± SD of three independent experiments. Plots show the mean ± SD from one representative experiment.

We observed an inhibition of at least 50% for all three transporters with butylated hydroxyanisole (BHA) and carnosic acid. In addition, ascorbyl palmitate (AP) inhibited OATP2B1, and lysine inhibited P-gp. Table 1 summarizes the effect of each of the antioxidants on the activity of the transporters. IC_50_ values for BHA were 71 ± 20 µM (OATP2B1), 206 ± 14 µM (BCRP), and 182 ± 49 µM (P-gp), and AP inhibited OATP2B1 with an IC_50_ value of 23 ± 10 µM. Pre-incubation of the OATP2B1-expressing cells with BHA and AP for 30 min did not further affect their inhibition potency (Table S2). Representative IC_50_ curves for each transporter are presented in Figure 3.

### 3.2. Comparison of AP and BHA IC_50_ Values with the Estimated Intestinal Concentrations

We compared the IC_50_ values of AP and BHA with their estimated intestinal concentrations to determine whether they may potentially interfere clinically with the transporters to affect oral bioavailability. As shown in Table 2, the I_gut_/IC_50_ ratios were less than 10 for both AP and BHA [30]. If I_gut_/IC_50_ was less than 10, the potential of the inhibitor to inhibit the transporter in vivo was considered low [31].

### 3.3. Effect of AP and BHA on OATP2B1-Mediated Valsartan Uptake

**Figure 4:**
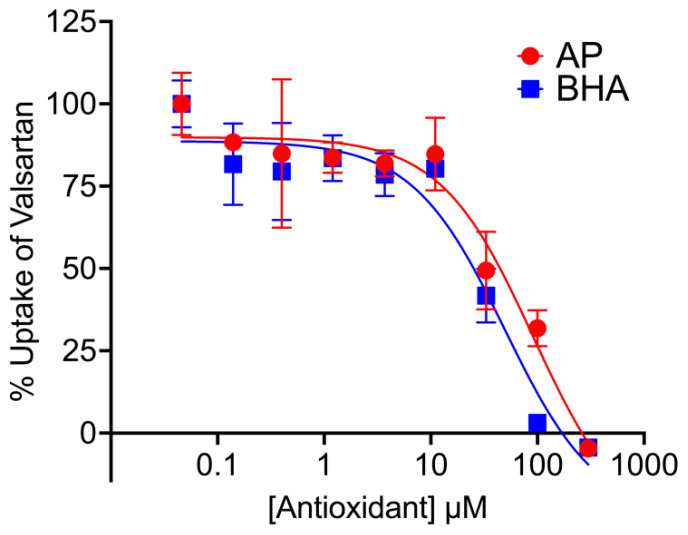
Concentration–response relationships for inhibition of OATP2B1-mediated valsartan transport by AP and BHA. Plots show mean ± SD from one representative experiment.

Numerous essential medicinal products faced recalls from the market due to the presence of N-nitrosamine impurities [10,32], and some of these medicinal products are substrates of the transporters that we investigated. A potential strategy used to address this issue involves incorporating antioxidants into the products to prevent N-nitrosamine formation due to degradation. We aimed to examine the effect of antioxidants on the transport of a drug substrate that had been subject to recalls due to the presence of N-nitrosamine impurities in certain lots [10]. Valsartan, a drug recognized as a substrate of OATPs [33], was chosen for this investigation. The average IC_50_ values for OATP2B1, derived from three independent experiments, were 25.5 ± 16.8 µM for AP and 19.1 ± 11.1 µM for BHA. Figure 4 represents data from one experiment. The calculated I_gut_/IC_50_ ratios for AP and BHA were 3.8 and 9.3, respectively, both below the threshold of 10 [30].

### 3.4. Effects of Elacridar or BHA on the Transport of Digoxin Efflux in Transwell Assays

**Figure 5:**
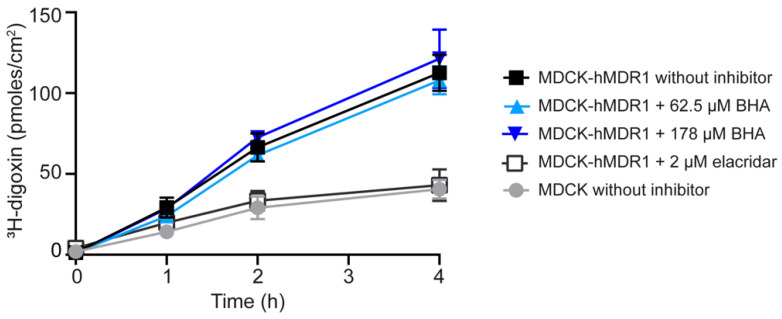
**Effects of elacridar or BHA on the efflux of digoxin by MDCK-hMDR1-cMDR1-KO cells.** The MDCK-hMDR1-cMDR1-KO cell line is a CRISPR-Cas9 engineered MDCK line that expresses human MDR1 while lacking the endogenous canine MDR1 (cABCB1). Elacridar (2 μM) or BHA (62.5 or 178 μM) was applied to both the basal and apical sides of the cells. The control cell line was MDCK, in which the canine MDR1 (cABCB1) has been knocked out. The efflux of ^3^H-digoxin (2.5 µM) from the basal to apical side was measured over a period of 4 h, both with and without the addition of the compounds to both sides. Data are presented as mean values ± SD from one representative experiment.

We evaluated the effect of BHA on inhibiting digoxin transport by P-gp using a transwell assay. The basal-to-apical flux of digoxin was approximately three- to five-fold higher in MDCK cells overexpressing human MDR1 compared to control cells that do not express canine or human MDR1. Treatment with elacridar (2 µM), a known P-gp inhibitor, diminished digoxin transport in the human MDR1-overexpressing cells to levels similar to those in the control cells, indicating the specific transport of digoxin by P-gp (Figure 5). The calculated maximal intestinal concentration of BHA (178 µM), assuming the ingestion of the maximum BHA dose (8 mg) from an oral formulation in 250 mL of intestinal fluid, was tested for its capability to inhibit P-gp. Neither the estimated maximal intestinal concentration of 178 µM nor a lower concentration (62.5 µM) inhibited digoxin transport (Figure 5). Notably, a higher concentration of BHA (250 µM) disrupted MDCK cell tight junctions as indicated by increased lucifer yellow permeability, precluding further assays to estimate P-gp inhibition by BHA.

## 4. Discussion

Antioxidants have potential as nitrite scavengers to mitigate N-nitrosamine formation in medicinal products [12,34]. In this study, we characterized the interactions of antioxidants used in oral formulations with three major intestinal transporters (OATP2B1, P-gp, and BCRP). Antioxidants are often added to oral pharmaceuticals to improve drug stability, but their potential impact on drug absorption by inhibiting these major intestinal drug transporters has not been extensively tested. Inhibition of these transporters can significantly alter drug bioavailability, as evidenced in clinical studies [20,21,22,23]. In the current work, we screened a panel of 30 molecular excipients with antioxidant properties to assess their ability to modulate the function of OATP2B1 using HEK-293 cells expressing this transporter. Additionally, we examined the effects of these excipients on BCRP and P-gp using membrane vesicles expressing each transporter. Carnosic acid and BHA emerged as significant inhibitors (≥50% inhibition) of the uptake of canonical substrates by all three transporters at a concentration of 200 µM. AP and lysine showed inhibitory activity only against OATP2B1 and P-gp, respectively. Although carnosic acid has been identified as a P-gp inhibitor in ATPase assays [35], carnosic acid was excluded from further consideration as the compound is not an FDA-approved inactive ingredient. Lysine was also not selected for further study, as lysine is a common ingredient in FDA-approved injectables rather than in oral dose formulations (https://www.accessdata.fda.gov/scripts/cder/iig/index.cfm, access date: 31 August 2022). To the best of our knowledge, this study was the first to evaluate the majority of these antioxidant excipients for their potential impact on major intestinal transporters. This study includes four antioxidants (i.e., (±)-α-tocopherol, butylated hydroxytoluene, L-ascorbic acid, and sodium thiosulfate) that were previously assessed and found not to inhibit these transporters [14,15,16]. Our findings provide data for the informed selection and evaluation of antioxidants in oral dosage forms.

To further evaluate the potential clinical relevance of BHA and AP, two antioxidants that interact with the three major intestinal transporters OATP2B1, BCRP, and P-gp, we estimated their maximal intestinal concentrations based on the maximum allowable oral amount set by the World Health Organization and the Code of Federal Regulations Title 21. According to the FDA’s guidance on the role of transporters in drug–drug interactions affecting intestinal absorption [30], excipients with I_gut_/IC_50_ ≥ 10 ([I_gut_], estimated maximal intestinal concentration) could potentially inhibit transporter-mediated drug absorption in clinical settings [30,31,36]. As shown in Table 2, the I_gut_/IC_50_ values for both BHA and AP are significantly lower than 10, suggesting minimal potential for the inhibition of OATP2B1, BCRP, and P-gp at the maximum expected concentration in the intestinal lumen. Furthermore, the I_gut_/IC_50_ ratios for BHA and AP, using valsartan as the drug substrate, were below the threshold of 10. Hence, it is unlikely that AP and BHA would significantly inhibit the OATP2B1-mediated uptake of valsartan clinically.

A crucial question remains as to whether the inhibition observed in inside-out vesicle assays for BCRP and P-gp translates to in vivo settings. To replicate the in vivo environment, we performed cellular efflux assays using BCRP- and P-gp-overexpressing MDCK cells, introducing the antioxidant into the culture medium. Notably, elacridar, a canonical P-gp inhibitor, effectively blocked digoxin efflux in our P-gp-overexpressing cell system, validating the suitability of a whole-cell system for P-gp inhibitor screening (Figure 5). In contrast to the vesicular assay, BHA did not impede digoxin transport in MDCK cells overexpressing P-gp, even at the maximal intestinal concentration of 178 μM. Additionally, we evaluated BHA’s interaction with BCRP using prazosin, a prototypical BCRP substrate, in MDCK cells. Similarly, 178 μM of BHA did not inhibit prazosin efflux in MDCK cells overexpressing BCRP, while Ko143, a BCRP inhibitor, significantly reduced prazosin transport (Figure S3).

The observed discrepancies between the results of the vesicular and cellular efflux assays could stem from several factors. First, cells, unlike single vesicles, are complex and integrated with a multitude of receptors, metabolic enzymes, and transporters. This intricate environment may hinder the effective penetration of BHA to the vicinity of intracellular BCRP and P-gp, potentially preventing the accumulation of sufficient concentrations to inhibit their transport activity. Furthermore, the substrates employed in the vesicular and cellular assays differ, potentially exhibiting distinct transport mechanisms. These structural variations among the substrates could influence their interactions with specific residues within the corresponding transporters, thereby affecting inhibitory potential. Therefore, caution should be exercised when extrapolating results from inside-out membrane vesicles to whole cells or in vivo situations.

In conclusion, the antioxidant BHA was identified as an inhibitor of the intestinal drug transporters OAPT2B1, BCRP, and P-gp. However, when comparing the IC_50_ values and the intestinal concentration of the antioxidant in the intestinal fluid, it suggests that the potential of BHA to inhibit the transporter in vivo was considered low for these transporters. Our findings provide data on the potential impact of antioxidants on the intestinal absorption and efflux of various drugs. Our results indicate that many antioxidants do not interact with OATP2B1, BCRP, and P-gp in cells, making them suitable candidates for use in oral dosage forms to inhibit the formation of N-nitrosamine impurities without compromising the bioavailability of co-administered drugs.

## Figures and Tables

**Table 1:** **Inhibition of transporter activity by antioxidants.** The uptake of a probe substrate was measured in the presence of an antioxidant (200 µM) or DMSO. The uptake from cells treated with DMSO was set as 100% and the uptake with the indicated antioxidants is presented as a percentage of this reference level. The values reported are the mean ± standard deviation (SD) from three replicates. Only ascorbyl palmitate (AP), butylated hydroxyanisole (BHA), carnosic acid, and lysine met the predetermined threshold for putative inhibitors of 50% inhibition. AP and BHA were selected for further evaluation because they are commonly utilized in oral drug formulations and are also included in the FDA’s Inactive Ingredients Database (IID).

Compounds		% Uptake of ^3^H-ES	% Uptake of ^3^H-CCK8	% Uptake of ^3^H-NMQ
		OATP2B1	BCRP	P-gp
**Antioxidants**	Ascorbic acid	103.6 ± 9.3	92.7 ± 5.9	113.6 ± 43.3
	Ascorbyl palmitate (AP)	28.3 ± 11.2	50.9 ± 9.9	86.9 ± 12.4
	Butylated hydroxytoluene (BH)	137.1 ± 34.8	102.8 ± 9.2	98.1 ± 20.3
	Cysteine hydrochloride	106.5 ± 14.8	90.0 ± 12.5	134.8 ± 41.1
	Propyl gallate	79.3 ± 17.2	86.5 ± 2.6	149.1 ± 67.6
	Vitamin E	83.5 ± 20.9	81.0 ± 12.6	67.9 ± 24
	3,4-dihydroxybenzoid acid	85.4 ± 4.4	78.5 ± 6.9	118.9 ± 20
	Anhydrous citric acid	128.8 ± 24.4	93.1 ± 6.7	92 ± 2.1
	Butylated hydroxyanisole (BHA)	19.8 ± 7.1	46.7 ± 5.5	42.1 ± 5.7
	Caffeic acid	119.8 ± 19.8	75.8 ± 3.7	133.8 ± 12
	Carnosic acid	18.8 ± 5.6	4.5 ± 4.8	0.0 ± 10.1
	Carnosine	119.3 ± 27.6	99.7 ± 11.6	82.6 ± 44
	Co-enzyme Q-10	116.1 ± 16.0	82.7 ± 16.2	90.3 ± 16.3
	EDTA (edetate disodium)	112.8 ± 25.2	110.4 ± 24.6	69.3 ± 20.5
	Ergothioneine	118.2 ± 30.6	112.5 ± 11.2	58.2 ± 23.6
	Erythorbic acid	123.3 ± 15.0	88.2 ± 7.5	81.8 ± 31.4
	Ferulic acid	136.4 ± 20.7	91.0 ± 4.7	100.4 ± 16.6
	Glutathione	107.9 ± 7.8	95.9 ± 2.2	79.8 ± 15.8
	Glycine	125.6 ± 23.0	99.7 ± 15.1	76.9 ± 4.1
	Histidine	108.9 ± 4.0	92.4 ± 1.6	87.6 ± 10.6
	Lysine	120.3 ± 21.4	109.0 ± 20.8	32.5 ± 44.3
	Methionine	113.2 ± 15.2	94.8 ± 9.4	86.7 ± 24.5
	Phosphoric acid	97.8 ± 5.0	83.0 ± 18.0	83.1 ± 27.6
	Potassium sorbate	98.8 ± 19.8	104.8 ± 14.5	103.5 ± 28.7
	Sesamol	103.1 ± 7.2	90.3 ± 3.7	121.3 ± 35.1
	Sodium bisulfite	90.1 ± 24.5	110.4 ± 9.3	96.1 ± 30.4
	Sodium metabisulfite	118.9 ± 31.7	94.5 ± 8.2	78.8 ± 17.5
	Sodium sulfite	92.9 ± 4.5	99.7 ± 5.5	77.7 ± 33.7
	Sodium thiosulfate, pentahydrate	104.4 ± 2.4	114.2 ± 22.8	77.4 ± 20
	Tartaric acid	101.1 ± 5.8	96.9 ± 16.6	108 ± 10.8
**Positive Control**	Cyclosporin A	83.3 ± 18.6	8.3 ± 4.5	0.0 ± 10.3
	Verapamil	194.2 ± 36.4	50.5 ± 6.8	0.0 ± 6
	Bromosulfophthalein	10.9 ± 8.1	0.3 ± 1.2	0.0 ± 22
	Erlotinib	10.5 ± 2.8	58.5 ± 3.9	63 ± 5
	Curcumin	8.2 ± 2.4	0.0 ± 1.8	0.0 ± 2.8

**Table 2:** **Comparison of IC_50_ values of the antioxidants with estimated intestinal concentrations.** (**a**). Estimated maximum concentrations of AP and BHA in intestinal fluid. (**b**). The ratio between the estimated intestinal concentration (I_gut_) of AP and BHA and the inhibition potencies (IC_50_) of the respective transporters. ND—not determined, as AP does not inhibit P-gp and BCRP by ≥50% at a concentration of 200 µM.

(**a**)				
**Compound**	**MW (g/mol)**	**FDA IID Oral Limit (mg/day)**	**% *w*/*w* in 500 mg Oral Unit**	**Max Intestinal Conc (µM) in 250 mL Fluid**
Ascorbyl palmitate (AP)	414.53	12	2% (10 mg)	**96.5**
Butylated hydroxyanisole (BHA)	180.24	8	1.6% (8 mg)	**178**
(**b**)				
Transporter	I_gut/_IC_50_			
	AP	BHA		
OATP2B1	4.2	2.5		
BCRP	ND	0.9		
P-gp	ND	1.0		

## Data Availability

The original contributions presented in the study are included in the article/Supplementary Materials, further inquiries can be directed to the corresponding author.

## Supplementary Materials

The following supporting information can be downloaded at: https://www.mdpi.com/article/10.3390/pharmaceutics16050647/s1, Figure S1: 3H-NMQ adsorbs on the filtration plate; Figure S2: Protein concentration per well was consistent; Figure S3: Effects of Ko 143 or BHA on the efflux of Prazosin by MDCK-hBCRP-cMDR1-KO cells; Table S1: Antioxidant screened for P-gp, BCRP and OATP2B1-mediated uptake inhibition; Table S2: Comparison of IC_50_ values obtained with and without preincubation of the OATP2B1 expressing cells with BHA and AP.
