# Supplementary material for: Effect of Antioxidants in Medicinal Products on Intestinal Drug Transporters
Source: Pharmaceutics. 2024 May 10;16(5):647. doi: 10.3390/pharmaceutics16050647 (PMC11124870; doi:10.3390/pharmaceutics16050647)
Supplement: Supplementary file 1 [file pharmaceutics-16-00647-s001.zip › pharmaceutics-2968426-supplementary.pdf]

Table S1. Antioxidant screened for P-gp, BCRP and OATP2B1-mediated uptake inhibition.

Name	MW	Source	Catalog Number	Structure (SMILES)	LogP	FDA IID oral limit (mg/day)	%w/w in 500mg oral unit	Max intestinal conc (uM) in 250mL fluid	Maximum Potency per unit dose	*FDA database available? (Y/N)	
3,4-dihydroxybenzoic acid	154.12	Sigma	37580-25G-F	C1=CC(=C(C=C1C(=O)O)O)O	0.86	--			--	N	
Ascorbic acid	176.12	Sigma	A92902-100G	C(C(C1C(=C(C(=O)O1)O)O)O)O	-1.85	600			20mg	Y	
Ascorbyl palmitate	414.5	Sigma	A1968-25G	CCCCCCCCCCCCCCCC(=O)OCC(C1C(=C(C(=O)O1)O)O)O	6	12	2	96.5	12mg	Y	
Butylated hydroxyanisole (2-tert-Butyl-4-hydroxyanisole)	180.24	Sigma	B1253-5G	CC(C)(C)C1=C(C=CC(=C1)O)OC	3.2	8	1.6	178	2.5mg/ml, 0.07mg	Y	
Butylated hydroxytoluene	220.35	Sigma	PHR1117-1G	CC1=CC(=C(C(=C1)C(C)(C)C)O)C(C)(C)C	5.1	3			0.24mg, 0.19mg/ml	Y	
Anhydrous citric acid	192.12	Sigma	C1909-25G	C(C(=O)O)C(CC(=O)O)(C(=O)O)O	-1.64	840			9.8mg, 26.03mg/ml, 0.09%w/v	Y	
Sodium metabisulfite	190.11	Fisher	S242-500	[O-]S(=O)S(=O)(=O)[O-].[Na+].[Na+]	-3.7	640			8mg, 2mg/ml	Y	
Propyl gallate	212.2	TCI Chemicals	G0018	CCCOC(=O)C1=CC(=C(C(=C1)O)O)O	1.8	7			0.07mg, 0.2mg/ml	Y	
Sodium thiosulfate, pentahydrate	248.19	Sigma	217247-25G	O.O.O.O.O.[O-]S(=O)(=S)[O-].[Na+].[Na+]	0.048				20mg, 22mg/ml	Y*	*pentahydrate not specfically listed
Vitamin E	430.7	Sigma	T3251	CC1=C(C2=C(CCC(O2)(C)CCCC(C)CCCC(C)CCCC(C)C)C(=C1O)C)C	12.2	85			300mg, 300mg/ml	Y*	*listed as "vitamin e polyethylene glycol succinate"
Carsonic acid	332.4	Sigma	SIAL-C0609-10MG	CC1=C(C2=C(CCC(O2)(C)CCCC(C)CCCC(C)CCCC(C)C)C(=C1O)C)C	4.9	--			--	N	
Erythorbic acid	176.12	SPECTRUM CHEMICALS & LABORATORY PRODUCTS	E1004-100GM	C(C(C1C(=C(C(=O)O1)O)O)O)O	-1.88	--			--	Y*	*listed but no info available
Caffeic acid	180.16	Sigma	C0625-2G	C1=CC(=C(C=C1C=CC(=O)O)O)O	1.15	--			--	N	
Ferulic acid	194.18	Thomas Scientific	C753K71	COC1=C(C=CC(=C1)C=CC(=O)O)O	1.51	--			--	N	

Glycine	75.07	Sigma	G7126	C(C(=O)O)N	-3.21	400	200mg	Y	
Lysine	146.19	Sigma	L5501-1G	C(CCN)CC(C(=O)O)N	-3.05	--	--	N*	*no oral forms
Histidine	155.15	Sigma	H3751	C1=C(NC=N1)CC(C(=O)O)N	-3.32		0.76mg, 5mg/ml		
Glutathione	307.33	Sigma	G4251-10G	C(CC(=O)NC(CS)C(=O)NCC(=O)O)C(C(=O)O)N	-6.4	--	--	N	
Co-enzyme Q-10	863.3	Millipore	C9538-100MG	CC1=C(C(=O)C(=C(C1=O)OC)OC)CC=C(C)CCC=C(C)CCC=C(C)CCC=C(C)CCC=C(C)CCC=C(C)CCC=C(C)CCC=C(C)CCC=C(C)CCC=C(C)C	10	--	--	N	
Carnosine	226.23	Sigma	C9625-10MG	C1=C(NC=N1)CC(C(=O)O)NC(=O)CCN	-4	--	--	N	
Ergothioneine	229.3	Sigma	E7521-5MG	C[N+](C)(C)C(CC1=CNC(=S)N1)C(=O)[O-]	0.3	--	--	N	
Sesamol	138.12	Sigma	S3003-5G-A	C1OC2=C(O1)C=C(C=C2)O	1.57	2325mg*	70% v/v*	N*	*sesame oil has an entry
Methionine	149.21	Sigma	M9625	CSCCC(C(=O)O)N	-1.87	--	--	Y*	*listed but no info available
Tartaric Acid	150.09	Sigma	T0250	C(C(C(=O)O)O)(C(=O)O)O	-0.76	1325	10mg	Y	
Phosphoric Acid	97.995	Millipore	466123-25G	OP(=O)(O)O	-2.1	19	6.4mg/5ml, 0.19mg	Y	
Cysteine hydrochloride	157.62	SPECTRUM CHEMICALS & LABORATORY PRODUCTS	C1473	C(C(C(=O)O)N)S.Cl	2.6	50		Y*	*anhydrous form
EDTA (edetate disodium)	336.21	Fisher	17892	C(C[NH+](CC(=O)[O-])CC(=O)[O-])[NH+](CC(=O)[O-])CC(=O)[O-].[Na+].[Na+]	-2.6		4mg	Y*	*of edetic acid
Sorbic acid	112.13	Sigma	S1626	CC=CC=CC(=O)O	1.33	80	0.94mg	Y	
Sodium bisulfite	104.06	Sigma	243973-100G	OS(=O)[O-].[Na+]	-1.2	9	1mg, 0.5mg/ml	Y	
Sodium sulfite	126.05	Sigma	207845	[O-]S(=O)[O-].[Na+].[Na+]	-4		5mg, 1.15mg/ml	Y	

Table S2. Comparison of IC_{50} values obtained with and without preincubation of the OATP2B1 expressing cells with BHA and AP. Pre-incubation of the OATP2B1 expressing cells with BHA and AP for 30 minutes did not significantly affect their inhibition potency. Mean ± SD of three independent experiments; a two-tailed t-test was applied.

	IC_{50} without preincubation	IC_{50} with preincubation	Significance
**Ascorbyl palmitate (AP)**	158.6 ± 65.9	95.0 ± 11.7	**No (p = 0.3886)**
**Butylated hydroxyanisole (BHA)**	76.9 ± 15.9	67.0 ± 12.8	**No (p=0.1801)**

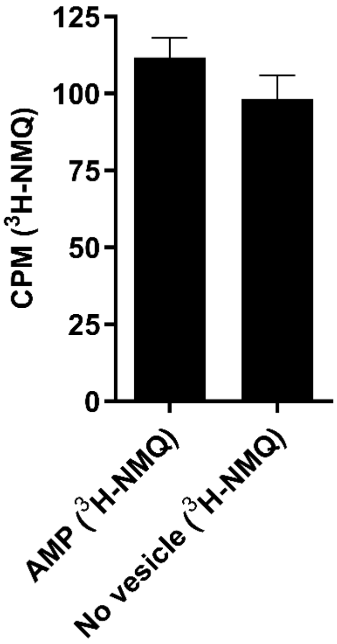

Figure S1. ^{3}H-NMQ adsorbs on the filtration plate. ^{3}H-NMQ was incubated with or without P-gp membrane vesicles (25 µg/well) in the presence of 5 mM AMP. Plates were incubated at 37 °C for 20 min. Each column represents the mean ± SD of counts per minute (CPM) of ^{3}H-NMQ uptake or adsorption.

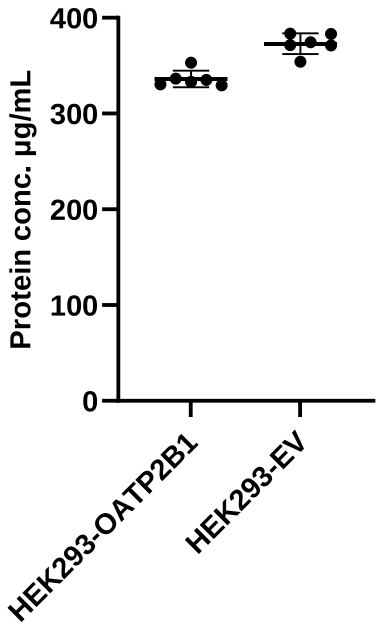

Figure S2. **Protein concentration per well was consistent.** Protein concentration was measured with BCA assay HEK-293 cells expressing OATP2B1 or empty vector. Each column represents the mean ± SD of protein concentration µg/mL.

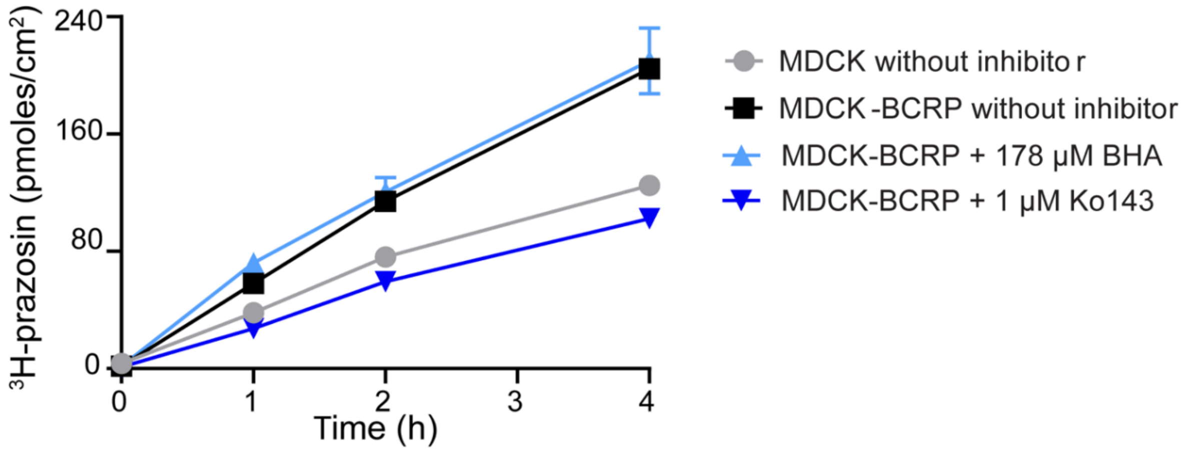

Figure S3. Effects of Ko 143 or BHA on the efflux of Prazosin by MDCK-hBCRP-cMDR1-KO cells. The MDCK-hBCRP-cMDR1-KO cell line is a CRISPR-Cas9 engineered MDCK line that expresses human BCRP while lacking the endogenous canine MDR1 (cABCB1). Ko143 (1 µM) or BHA (178 µM) was applied to both basal and apical sides of the cells. The control cell line used was MDCK, in which canine MDR1 (cABCB1) has been knocked out. The efflux of ^{3}H-prazosin (1 µM) from the basal to apical side was monitored over a period of 4-hours, both with and without the addition of the compounds to both sides. Data are presented as mean values ± SD from one representative experiment.
